# Diagnostic value of [^68^Ga]Ga-FAPI-46 PET in comparison to [^18^F]FDG PET in triple-negative breast cancer and potential for theranostic application

**DOI:** 10.1038/s41598-026-61253-8

**Published:** 2026-08-03

**Authors:** Alina Toni Küper, Ieva Ciuciulkaite, Anja Welt, Sofia Carrilho Vaz, Ilektra Antonia Mavroeidi, Rainer Hamacher, Tristan Demmert, Kim Magaly Pabst, Alexandros Moraitis, Loic Djaileb, Robert Seifert, Lale Umutlu, Martin Schuler, Ken Herrmann, Wolfgang Peter Fendler, Sherko Kümmel, David Kersting

**Affiliations:** 1 Department of Nuclear Medicine and German Cancer Consortium (DKTK), University Hospital Essen, University of Duisburg-Essen, Hufelandstrasse 55, Essen, 45147 Germany; 2 Department of Medical Oncology, West German Cancer Center, University Hospital Essen, University of Duisburg-Essen, Essen, Germany; 3 National Center for Tumor Diseases (NCT), NCT West, Essen, Germany; 4 Department of Nuclear Medicine and Radiopharmacology, Champalimaud Clinical Center, Champalimaud Foundation, Lisbon, Portugal; 5 Department of Radiology, Leiden University Medical Center, Leiden, The Netherlands; 6 Nuclear Medicine Department, LRB, CHU Grenoble Alpes, INSERM, Université Grenoble Alpes, Grenoble, France; 7 Department of Nuclear Medicine, University Hospital Bern, University of Bern, Bern, Switzerland; 8 Institute of Interventional and Diagnostic Radiology and Neuroradiology, University Hospital Essen, University of Duisburg-Essen, Essen, Germany; 9 Breast Unit Essen, Kliniken Essen-Mitte, Essen, Germany

**Keywords:** Triple-negative breast cancer, Molecular imaging, PET, FAPI, Theranostics, Cancer, Oncology

## Abstract

This single-center subgroup analysis of a prospective trial (NCT04571086) at University Hospital Essen investigated tumor uptake and detection rates of [^68^Ga]Ga-FAPI-46- versus [^18^F]FDG PET in triple-negative breast cancer (TNBC) patients undergoing initial or follow-up staging. Lesions were recorded across anatomical regions, with detection efficacy, uptake values, tumor-to-liver ratios, and tumor volumes assessed. 22 patients were included (initial- *n* = 10, follow-up staging *n* = 12). No significant difference in region-based detection rates was observed between [^18^F]FDG- (93%, 53/57 regions) and [^68^Ga]Ga-FAPI-46 PET (93%, 53/57 regions) (McNemar *p* = 1.0). Three patients (25%) at follow-up staging showed an average tumor SUV_mean_ ≥5 on [^68^Ga]Ga-FAPI-46 PET; 2/3 showed SUV_max_ >10 in most lesions. Mean tumor volume was higher for [^68^Ga]Ga-FAPI-46, though not statistically significant at initial- (50.1 ± 120.8 mL vs. 11.9 ± 18.5 mL) and follow-up staging (102.2 ± 122.3 mL vs. 79.0 ± 90.4 mL). [⁶⁸Ga]Ga-FAPI-46 PET demonstrated comparable detection in TNBC; high tumor uptake in a subset, supporting its theranostic potential.

**Supplementary Information:**

The online version contains supplementary material available at 10.1038/s41598-026-61253-8.

## Introduction

Breast cancer ranks second among cancer-related causes of death in women, despite great advances in therapy options^1^. Triple negative breast cancer (TNBC) corresponds to 10-15% of all breast cancer subtypes, often presents with visceral metastases, and shows high [^18^F]FDG avidity^2,3^.

Current [^18^F]FDG positron emission tomography (PET) comes with limitations for the staging of patients with breast cancer, therefore it is recommended in current guidelines only on selected occasions^4–6^. These shortcomings include detection limits for small tumors, micrometastases, hormone receptor-positive carcinomas and lobular subtype, as well as false positive findings after biopsy or for nonmalignant breast pathologies including infections, fibroadenomas, and ductal adenomas^3,7^.

PET imaging using novel radioligands may overcome these limitations. Fibroblast activation protein alpha (FAP) has emerged as novel pan-tumor target for radiotheranostics in many types of solid cancers^8^. FAP is highly overexpressed on the membrane of cancer associated fibroblasts (CAFs), the most abundant component of the tumor microenvironment^9^. Although, the first use of FAPI PET in patients with BC was described early after introduction of this imaging modality, the number of studies on FAPI PET in breast cancer is yet limited^9,10^ particularly in patients with TNBC. Nevertheless, FAP-targeted radiotheranostics are promising, as FAP was described to be a solid stromal cell marker of all breast cancer subtypes; in a metanalysis FAP expression ranged between 50% and 100% of analyzed samples^11^. Moreover, initial reports describe a more favorable tumor-to-background ratio and higher sensitivity for FAPI PET in comparison to [^18^F]FDG PET^12^.

We hypothesized that [^68^Ga]Ga-FAPI-46 PET/CT allows accurate tumor detection in patients with TNBC. In this study, we compare diagnostic performances of [^68^Ga]Ga-FAPI-46 and [^18^F]FDG PET/CT in patients with TNBC. Our aim was to evaluate tumor detection rates and tracer uptake, with a special focus on analyzing the potential for theranostic applications.

## Materials and methods

### Patients and ethics

This is a subanalysis of the prospective University Hospital Essen FAPI PET observational trial (NCT04571086), which was initiated in April 2020. All patients with TNBC who underwent whole-body [^18^F]FDG and [^68^Ga]Ga-FAPI-46 PET/CT under clinical indication for initial diagnostic workup or follow-up staging (e.g. restaging for treatment response assessment, suspected local recurrence or metastatic disease [detailed indications are provided in ***Supplementary Table ******S1****]*) were selected from the study cohort. [^18^F]FDG and [^68^Ga]Ga-FAPI-46 PET/CT were performed within an interval of 5 days, without relevant CT-morphological changes. Clinical baseline data were collected from electronic patient records. For patients at primary staging, the Combined Positive Score (CPS) was utilized to assess Programmed death-ligand 1 (PD-L1) expression if available in clinical records. Written informed consent for study participation and to undergo clinical PET examinations were obtained. All investigations were conducted in accordance with the Declaration of Helsinki and national regulations. The local institutional ethics committee (University of Duisburg–Essen, medical faculty) approved the study (ethics protocol permits 19-8991-BO and 20–9485-BO).

### [^68^Ga]Ga-FAPI-46 PET/CT imaging

PET/CT images were acquired on a Biograph Vision 600 or a Biograph mCT PET/CT system (both Siemens Healthineers, Erlangen, Germany). The mean [^68^Ga]Ga-FAPI-46 administered activity was 119 MBq and the mean uptake time was 20 min. Details on imaging protocols are given in supplemental material.

### Study endpoints

Primary endpoints were detection efficacy on a per region and per patient level. Secondary endpoints were analyses of tumor volumes, lesion-based detectability, tumor uptake characteristics, and their correlation with PD-L1 expression.

### PET image analysis

All PET images were analyzed by 3 nuclear medicine physicians with several years of experience in PET reporting. Readers were not blinded to clinical information or to the results of the corresponding alternative PET scan. When findings were discrepant, images were re-evaluated and a final decision was reached by majority consensus. For patient-based and region-based analysis, [^68^Ga]Ga-FAPI-46 PET- or [^18^F]FDG PET-positive lesions were reported for each patient separately. Six different anatomic categories derived from the TNM classification for breast cancer^13^ were defined: primary tumor, regional lymph node metastases, distant lymph node metastases, liver metastases, bone metastases and other distant metastases. [^68^Ga]Ga-FAPI-46/[^18^F]FDG positivity was defined as visually markedly increased lesion uptake compared with local background. Further rules for lesion- and region-based detection are given in the Supplement.

### Lesion analysis and quantification

Semi-automatic segmentation of all [^68^Ga]Ga-FAPI-46 PET and/or [^18^F]FDG PET-positive lesions per patients was performed using Syngo.via software (Siemens Healthineers) to record SUV_mean_, total number of lesions, and total tumor volume in a PERCIST-like approach^14^. Lesion boundaries were determined using a 41% isocontour^15^. Details are given in supplemental material.

Moreover, the number of patients showing and SUV_max_ ≥10 in the majority of tumor lesions, which is used at our center to select patients for radionuclide therapy, was estimated^16^.

For an analysis of uptake with a focus on the region-level, the mean SUV_max_ of the hottest lesion in each of the predefined anatomic regions was determined for [^68^Ga]Ga-FAPI-46- and [^18^F]FDG, separately.

### Statistics

All statistical analyses were conducted using R software version 4.3.2 (R Foundation for Statistical Computing, Vienna, Austria). Mean values are presented as mean ± standard deviation (SD). To compare the detection accuracy on the region level, a McNemar test was employed. A paired t-test was used to compare the mean tumor volume, average tumor SUV_mean_, and average tumor TLR_mean_ (TLR_mean_= tumor SUV_mean_/liver SUV_mean_) per patient, as well as the total number of the detected lesions. Beforehand, the Shapiro-Wilk test was applied to verify normal distribution of data. The Pearson correlation coefficient was used to correlate the SUV_max_ of the primary tumor with the histopathologic CPS value. For all tests P-values (P) < 0.05 were considered statistically significant. The graphical abstract was designed using BioRender.com.

## Results

### Patient and imaging characteristics

Ten patients underwent [^18^F]FDG and [^68^Ga]Ga-FAPI-46 PET for initial staging and twelve patients for follow-up. One patient was tumor-free on follow-up imaging and excluded from further data analyses. Detailed patient characteristics are presented in ***Table*** ***1*** and* Supplementary Table ****S1***. Two patients at primary staging had undergone neo-adjuvant chemotherapy (CTx) and eight were therapy-naïve. The CPS, evaluating PD-L1 expression, was available in 50% of patients at primary staging.

**Table 1:** Baseline patient characteristics.

Patients	Initial Staging	Follow-up staging
	10	12
Age (y)	52 (30-83)	47 (37-69)
Histologic Subtypes		
ductal	9 (90%)	6 (50%)
lobular	0 (0%)	0 (0%)
unknown	1 (10%)	6 (50%)
Grade		
1	0	0
2	0	0
3	10	12
UICC at diagnosis		
I	3 (30%)	3 (25%)
IIA	3 (30%)	3 (25%)
IIB	2 (20%)	1 (8%)
IIIA	0 (0%)	2 (17%)
IIIB	0	0
IIIC	0	1 (8%)
IV	2 (20%)	1 (8%)
unknown	0	1 (8%)
M1 at staging	1 (10%)	9 (75%)
BRCA positive	3 (30%)	1 (8%)
BRCA tested	10 (100%)	10 (83%)
**Prior therapy lines**		
Breast surgery	0 (0%)	11 (92%)
RT	0 (0%)	6 (50%)
CT	2 (20%)	11 (92%)
Targeted therapy	0 (0%)	6 (50%)

BRCA: Breast Cancer gene; CT: Chemotherapy; RT: Radiotherapy; UICC: Union for International Cancer Control.

Most patients (*n* = 14, 64%) underwent both scans on the same day. Five patients (23%) received [^18^F]FDG PET before [^68^Ga]Ga-FAPI-46 PET, while three patients (14%) received [^68^Ga]Ga-FAPI-46 PET first. The interval between [^18^F]FDG PET and [^68^Ga]Ga-FAPI-46 PET range − 2 to + 5 days.

### Detection rates

In the patient-based analysis, all patients at initial staging (*n* = 10, 100%) showed [^68^Ga]Ga-FAPI-46 PET- and [^18^F]FDG PET-positive tumors (at least one lesion per patient). In the follow-up staging group, 11 patients (92%) presented with tumor lesions, and all these patients (*n* = 11, 100%) showed [^68^Ga]Ga-FAPI-46 PET- and [^18^F]FDG PET -positive tumors. Across all tumor-affected regions (encompassing both patients at initial staging or follow-up staging) no significant difference between region-based detection rates for [^18^F]FDG PET (93%, 53/57 regions) and [^68^Ga]Ga-FAPI-46 PET (93%, 53/57 regions) was observed (McNemar *p* = 1.0).

**Fig. 1:**
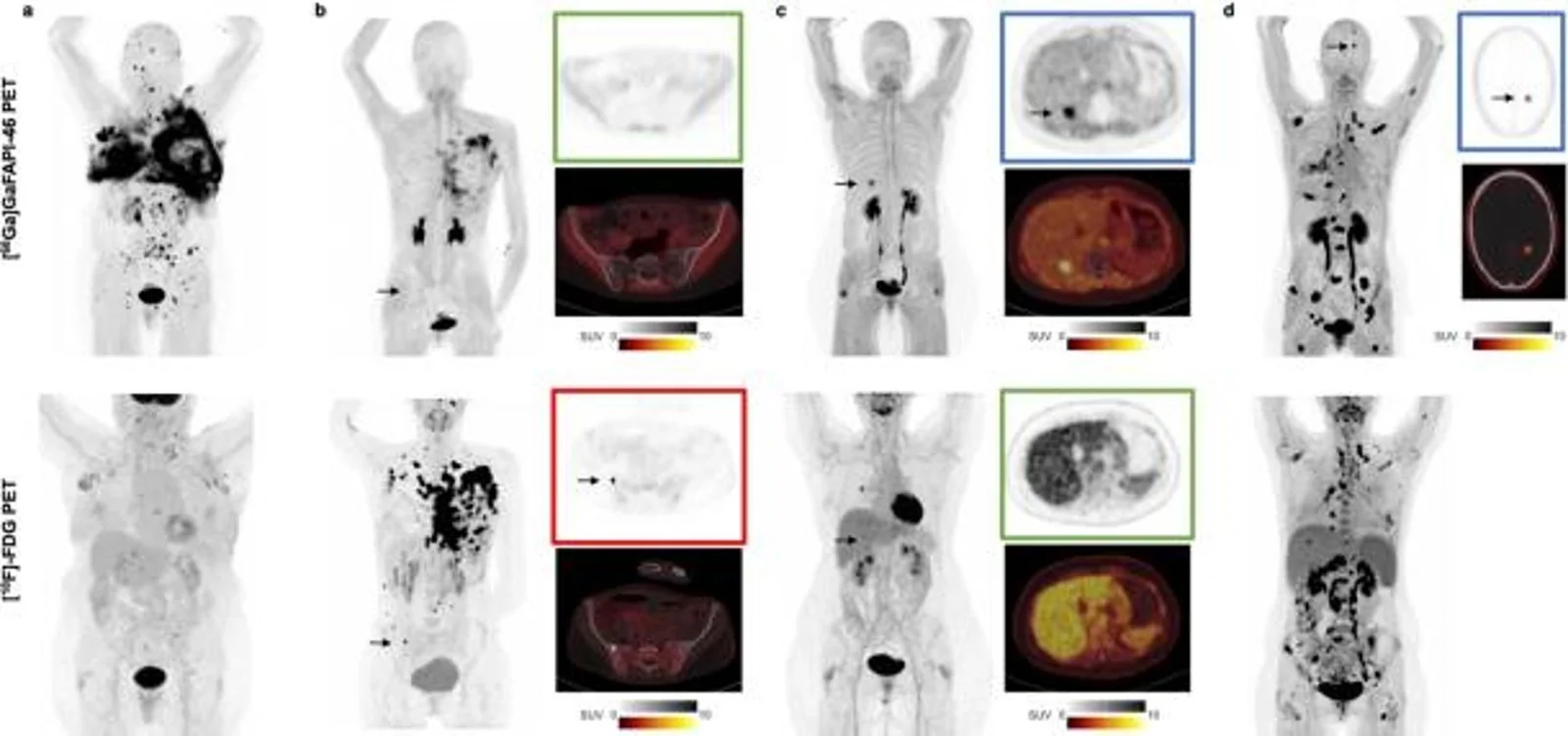
Image examples for varying diagnostic performance of [^68^Ga]Ga-FAPI-46 and [^18^F]FDG. PET maximum-intensity projection of a patient with cutaneous involvement of breast cancer extending from both breasts to the left shoulder, visible only on [^68^Ga]Ga-FAPI-46 PET (**a**). Each further panel shows maximum-intensity projection (left), transversal PET (top right) and transversal fused PET/CT images (bottom right). Bone metastasis, that was only visible on [^18^F]FDG PET, along with a more extensive visceral involvement (**b**). Liver metastasis that was detected only by [^68^Ga]Ga-FAPI-46 PET (**c**). Cerebral metastasis detected using [^68^Ga]Ga-FAPI-46 PET (**d**). Mean SUV_max_ uptake was defined as low ≤ 5 (green outline), intermediate 5 ≤ SUV_max_ ≤ 10 (blue outline) and high ≥ 10 (red outline) and refers to region-based mean SUV_max_ (mean of all [^68^Ga]Ga-FAPI-46/[^18^F]FDG positive regions per patient). CT: Computer tomography; FAPI: Fibroblast activation protein inhibitor; FDG: Fluorodesoxyglukose; PET: Positron-emission-tomography; SUV: Standardized uptake value.

Patients at initial staging showed a total of 23 tumor regions that were detected by either [^68^Ga]Ga-FAPI-46 PET or [^18^F]FDG PET. Region-based detection rates were 91% (21/23 detected regions) for [^68^Ga]Ga-FAPI-46 PET and 87% (20/23) for [^18^F]FDG PET. ***Figure ******1******a*** shows a patient example of extensive cutaneous involvement of breast cancer which was positive on [^68^Ga]Ga-FAPI-46 PET but negative on [^18^F]FDG PET.

In patients at follow-up staging, a total of 34 tumor regions were observed. Region-based detection rates were 94% (32/34 detected regions) for [^68^Ga]Ga-FAPI-46 PET and 97% (33/34) for [^18^F]FDG PET. ***Figure ******1******b, c, d*** show patient examples of discrepant or notable PET findings.

Details of the region-based analysis are presented in ***Table ******2***.

**Table 2:** Region-Based [^68^Ga]Ga-FAPI-46 and [^18^F]-FDG Uptake.

Initial Staging (10 patients)									
Region	n			SUV_max_		SUV_peak_		TLR_peak_	
	total	FAPI	FDG	FAPI	FDG	FAPI	FDG	FAPI	FDG
Breast tumor	10	10 (100%)	9 (90%)	13.5 ± 4.2	17.2 ± 8.8	9.7 ± 2.6	12.1 ± 8.3	9.4 ± 3.0	6.3 ± 7.3
Regional lymph node metastases	8	6 (60%)	8 (80%)	6.1 ± 5.2	7.0 ± 4.9	4.4 ± 3.9	3.9 ± 2.3	4.7 ± 5.6	1.8 ± 1.0
Distant lymph node metastases	1	1 (10%)	1 (10%)	5.8	5.7	4.8	3.3	6.9	1.3
Liver metastases	1	1 (10%)	0	6.8	0	4.6	0	6.6	1.4 ± 0.3
Bone metastases	2	2 (20%)	2 (20%)	11.9 ± 9.1	7.5 ± 3.0	6.7 ± 5.9	3.6 ± 1.0	8.8 ± 9.4	0
Other metastases	1	1 (10%)	0	25.3	0	17.3	0	24.7 ± 0	0
Follow-up staging (11 patients)									
Breast (primary/relapse)	6	6 (55%)	6 (55%)	7.1 ± 2.6	14.0 ± 15.1	5.2 ± 2.0	8.3 ± 8.6	5.3 ± 2.4	6.0 ± 6.7
Regional lymph node metastases	8	8 (73%)	8 (73%)	8.7 ± 5.4	16.4 ± 9.2	6.3 ± 4.4	9.7 ± 6.7	5.8 ± 3.1	6.3 ± 6.0
Distant lymph node metastases	6	6 (55%)	6 (55%)	8.5 ± 3.7	17.2.8 ± 8.5	6.6 ± 3.3	8.9 ± 4.6	6.7 ± 5.3	7.1 ± 8.4
Liver metastases	4	3 (27%)	3 (27%)	4.1 ± 2.9	6.9 ± 3.7	3.2 ± 2.3	3.9 ± 1.3	2.9 ± 1.3	3.8 ± 3.3
Bone metastases	3	2 (18%)	3 (27%)	21.9 ± 11.5	14.1 ± 4.5	18.4 ± 9.2	7.4 ± 4.1	21.2 ± 2.0	5.9 ± 7.5
Other metastases	7	7 (64%)	7 (64%)	9.4 ± 6.4	11.5 ± 8.3	5.6 ± 3.8	7.1 ± 6.3	5.1 ± 3.0	5.4 ± 6.8

Data are presented as mean ± SD; FAPI: Fibroblast activation protein inhibitor; FDG: Fluorodesoxyglukose SUV: Standardized uptake value; TLR: Tumor to liver ratio (TLRpeak = tumor SUVpeak/liver SUVpeak).

### Lesion-based analysis and tumor volumes

**Fig. 2:**
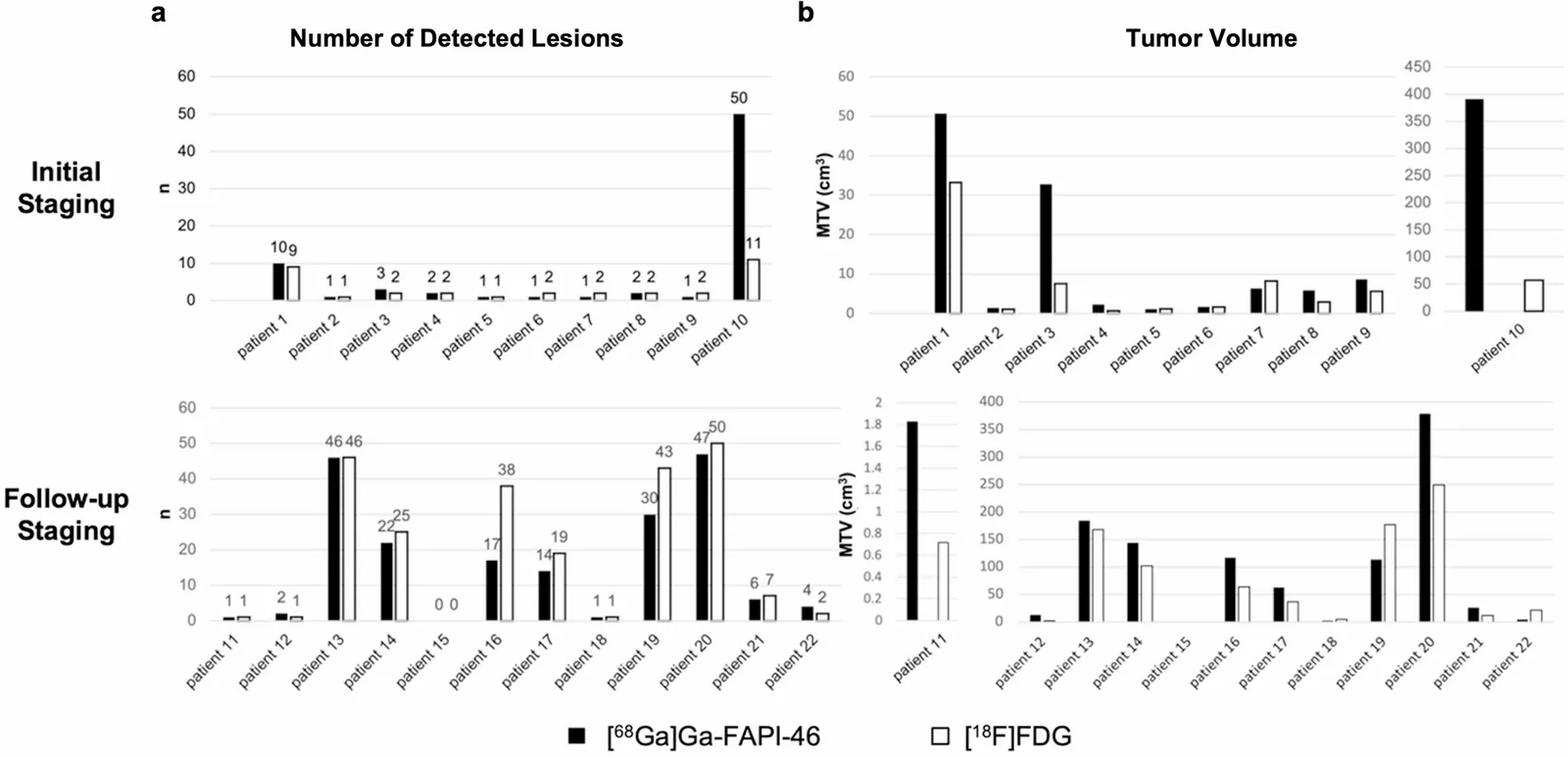
Comparison of number of detected lesions (**a**) and tumor volumes (**b**) between [^68^Ga]Ga-FAPI-46 and [^18^F]FDG PET/CT. Note: Patient 10 and 11 present different tumor volume scales to better include the results of an extremely high and low MTV, respectively. FAPI: Fibroblast activation protein inhibitor; FDG: Fluorodesoxyglukose.

In patients at primary staging, a total of *n* = 72 [^68^Ga]Ga-FAPI-46-positive lesions were identified, with a total tumor volume of 506.2 mL, compared to *n* = 34 [^18^F]FDG-positive lesions with a total tumor volume of 119.0 mL (***Fig. ******2******a and b***). Accordingly, the mean number of detected lesions per patient was higher for [^68^Ga]Ga-FAPI-46 PET (7.2 ± 15.2 vs. 3.4 ± 3.5 for [^18^F]FDG PET (*p* = 0.4), however, the difference was not statistically significant. These results are dominated by a single patient with 50 [^68^Ga]Ga-FAPI-46-positive but only 11 [^18^F]FDG-positive lesions (see patient example in ***Fig. ******1******a***).

**Fig. 3:**
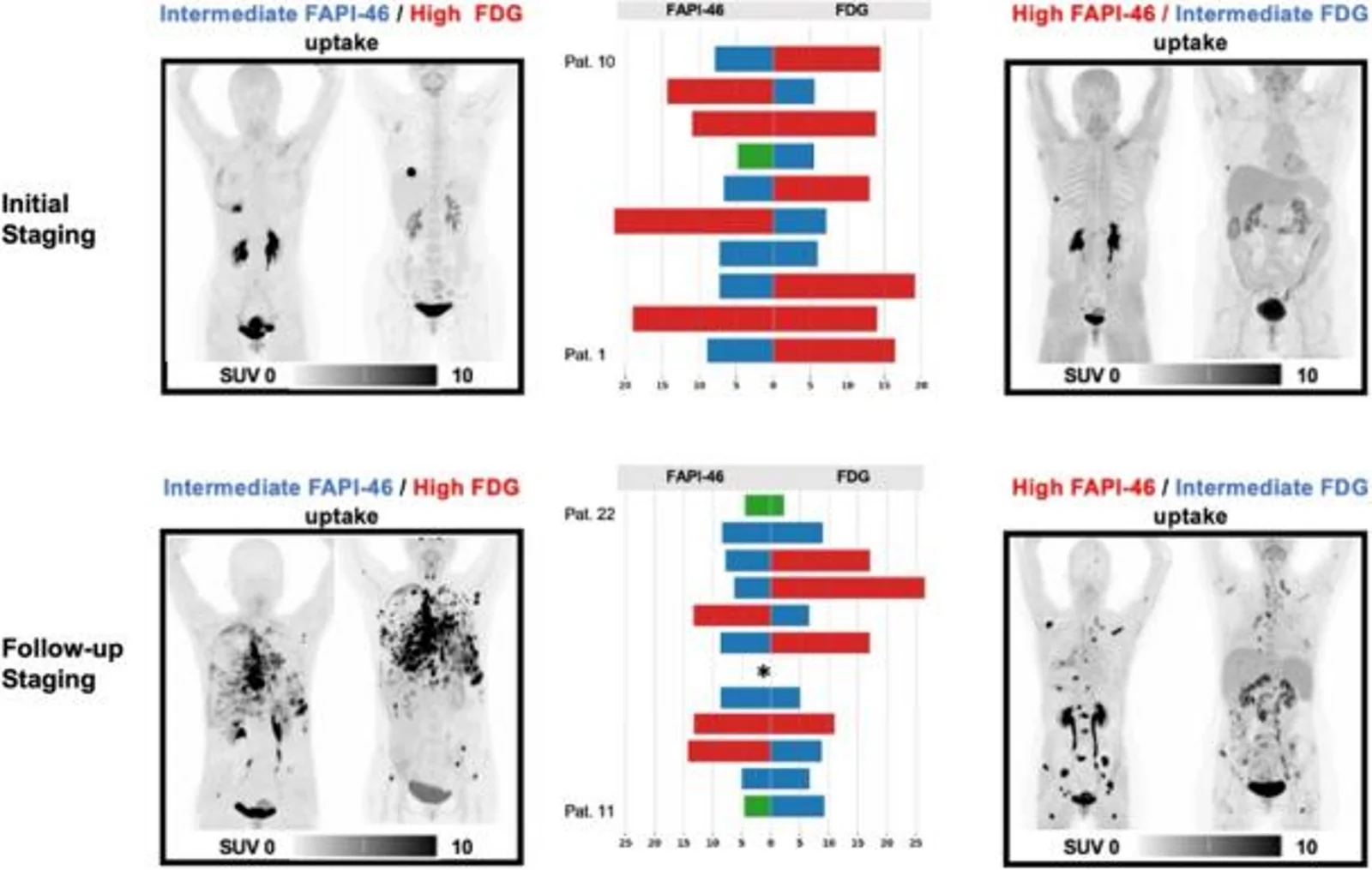
Patient-based comparison of [^68^Ga]Ga-FAPI-46 (FAPI-46) and [^18^F]FDG (FDG) uptake. Image examples (maximum intensity projection) of patient groups show different patterns of [^68^Ga]Ga-FAPI-46 and [^18^F]FDG uptake. Mean SUV_max_ uptake was defined as low ≤ 5 (green), intermediate 5 ≤ SUV_max_ ≤ 10 (blue) and high ≥ 10 (red) and refers to region-based mean SUV_max_ (mean of all [^68^Ga]Ga-FAPI-46/[^18^F]FDG positive regions per patient). * One patient showed no tumor burden at the time of PET imaging.

In patients at follow-up staging, a total of *n* = 192 [^68^Ga]Ga-FAPI-46-positive lesions with a total tumor volume of 1045.4 mL were identified, compared to *n* = 233 (833.9 mL) [^18^F]FDG-positive lesions (***Fig. ***2***a ***and **b**). The mean number of detected PET lesions per patient was not significantly different for [^18^F]FDG (*n* = 17.8 ± 18.7 detected lesions) compared to [^68^Ga]Ga-FAPI-46 (*n* = 15.1 ± 15.9; *p* = 0.79).

Without being statistically significant, the mean tumor volume in patients at initial staging was higher for [^68^Ga]Ga-FAPI-46 PET compared to [^18^F]FDG PET (50.1 ± 120.8 mL vs. 11.9 ± 18.5 mL). Similar findings were observed in follow-up staging ([^68^Ga]Ga-FAPI-46: 102.2 ± 122.3 ml vs. [^18^F]FDG: 79.0 ± 90.4 ml).

### Tumor uptake and correlation with PD-L1 expression

In initial staging, the average tumor SUV_mean_ and TLR_mean_ per patient were not significantly different between [^68^Ga]Ga-FAPI-46 and [^18^F]FDG PET (average tumor SUV_mean_ 7.4 ± 2.7 vs. 8.0 ± 3.6, *p* = 0.7; and TLR_mean_ 7.3 ± 3.1 vs. 3.8 ± 2.6, *p* = 0.05). In follow-up staging, comparable results were observed (average tumor SUV_mean_ 4.9 ± 1.9 vs. 6.3 ± 3.7, *p* = 0.2; and TLR_mean_ 4.7 ± 1.7 vs. 3.7 ± 3.4, *p* = 0.3).

To analyze the individual variability in tumor uptake between [^68^Ga]Ga-FAPI-46 and [^18^F]FDG PET with a focus on the region-level, the mean SUV_max_ of the hottest lesion per PET-positive region in each patient was compared. The results of this analysis are presented in ***Fig. ***3 illustrating the individual heterogeneity in region-based uptake. In some patients, particularly at initial staging, low uptake in [^68^Ga]Ga-FAPI-46 PET was accompanied by high uptake in [^18^F]FDG PET and vice versa; however, no general trend was observable. Boxplot representations of mean SUV_max_ values from tumor-affected regions comparing [⁶⁸Ga]Ga-FAPI-46 and [¹⁸F]FDG PET uptake, shown separately for initial and follow-up staging, are provided in ***Fig. ******S1***.

A strong inverse correlation was observed between [^68^Ga]Ga-FAPI-46 uptake (SUV_max_) of the primary tumour and the CPS score (Spearman ρ = −0.90, *p* = 0.04). In contrast, no significant correlation was found between [^18^F]FDG uptake and CPS score (Spearman ρ = 0.40, *p* = 0.51).

**Fig. 4:**
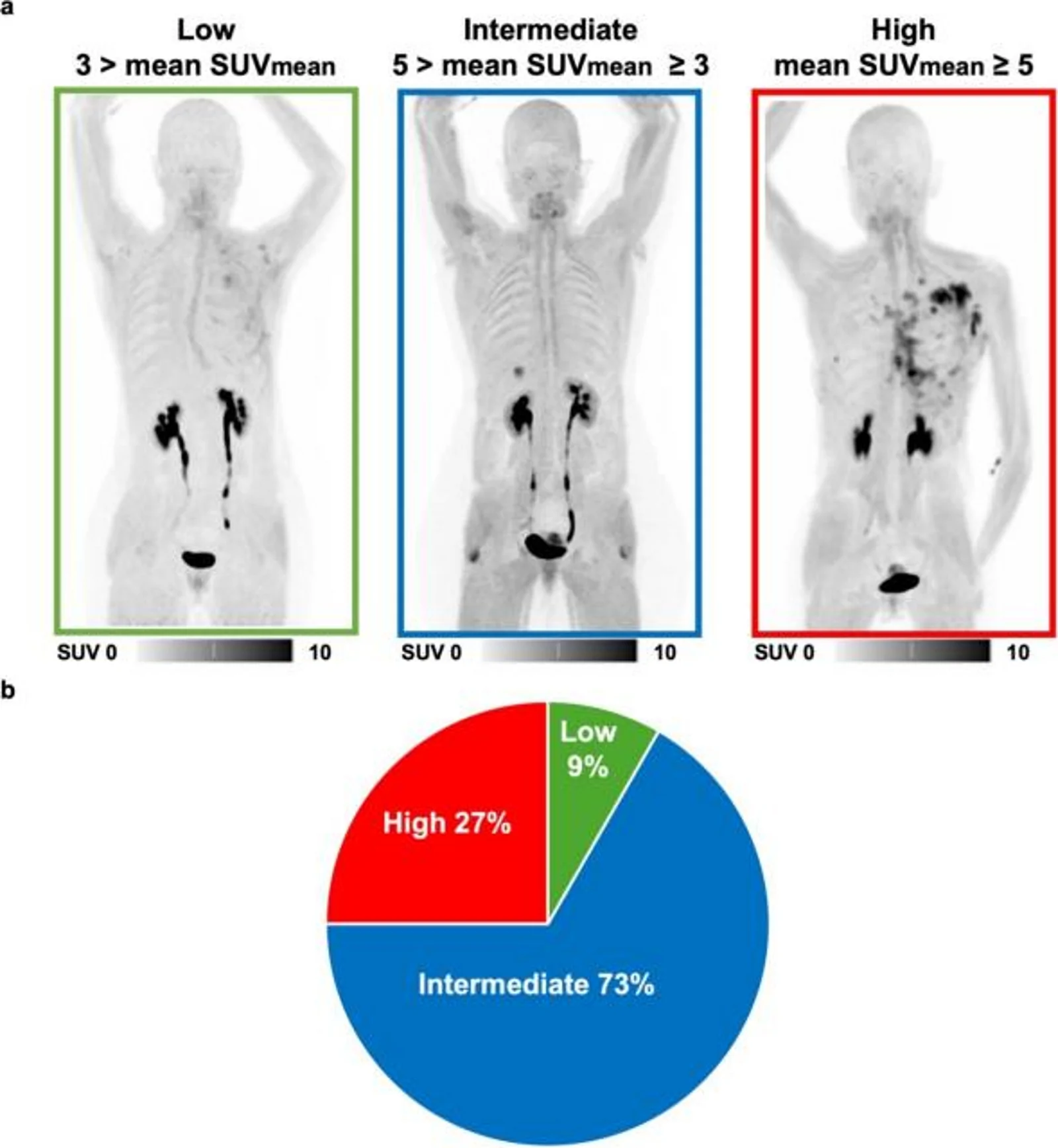
Image examples (maximum intensity projection) of patients with low, intermediate, high uptake (**a**) and distribution of average tumor [^68^Ga]Ga-FAPI-46 SUV_mean_ uptake in patients evaluated for follow-up (**b**). Arm positioning was adapted to patient mobility, with arms preferably positioned overhead.

Two evaluate the potential for FAP-directed radioligand therapy, we first grouped patients at follow-up staging according to their average tumor SUV_mean_ and TLR_mean_. 25% patients showed a mean global tumor SUV_mean_ > 5 (***Fig***. ***4***). All patients presented with an average TLR_mean_ >1, 90.9% with an average TLR_mean_ >3 and 63.6% with an average TLR_mean_ > 5. Moreover, 2 patients at follow-up staging (17%) showed a SUV_max_ >10 in ≥ 50% of tumour lesions, a criterion that is used at our center to clinically select patients who are eligible for [^90^Y]Y-FAPI-46 radioligand therapy.

## Discussion

To our best knowledge, this is the largest head-to-head comparison of [^68^Ga]Ga-FAPI-46 and [^18^F]FDG PET in patients with TNBC published so far. Although [^18^F]FDG PET is known to have high diagnostic accuracy in TNBC, the equal diagnostic quality of [^68^Ga]Ga-FAPI-46 PET highlights its applicability for all breast cancer subtypes, with its well-known strength for identification of distant metastases^9,17,18^. Considering that our analysis of patient- and region-based detection rates did not show significant differences between [^68^Ga]Ga-FAPI-46 and [^18^F]FDG PET (***Table ******2***), [^68^Ga]Ga-FAPI-46 PET may be considered as a complementary tool to [^18^F]FDG PET for initial- and follow-up staging of patients with this disease. By providing additional biological information on the tumour microenvironment, FAPI PET may enable extended tumour characterisation and potentially support radiotherapy planning. In detailed analysis, for both modalities additional tumor lesions were detected (one additional breast tumor and three additional distant metastases for [^68^Ga]Ga-FAPI-46 versus two additional regional lymph node metastases and two additional distant metastases for [^18^F]FDG PET, (***Fig. ******1***). Moreover, the comparison of region-based tumor uptake revealed an intra- and interindividual heterogeneity without uniform correlation between [^68^Ga]Ga-FAPI-46 and [^18^F]FDG uptake (***Fig. ******3***). These data indicate that [^68^Ga]Ga-FAPI-46 can provide additional information and might complementarily be used.

One of the advantages of [^68^Ga]Ga-FAPI-46 PET is low background activity in areas with high physiological [^18^F]FDG uptake like liver tissue^19,20^. In two patients, additional liver metastases were detected in [^68^Ga]Ga-FAPI-46 PET whereas in one patient an additional liver metastasis was detected in [^18^F]FDG PET (***Fig. ******1******c***). Moreover, one patient showed a clearly discernible brain metastasis with low background uptake in [^68^Ga]Ga-FAPI-46 PET (***Fig. ******1******d***), that was not included into the scan area of the corresponding [^18^F]FDG PET.

The low background activity in the liver also leads to higher TLR values in [^68^Ga]Ga-FAPI-46 PET. However, the differences were not statistically significant and did not translate into higher detection rates. This contrasts with previous studies which describe an increased number of detected tumor regions in [^68^Ga]Ga-FAPI^19,21,22^. However, these studies did not include patients with TNBC but focused on other breast cancer subtypes. An additional benefit of [^68^Ga]Ga-FAPI-46 PET is its simple scanning protocol, enhancing patient comfort with minimal need for preparation, no fasting or antidiabetic adjustments, and short 10-minute uptake times^23^. Furthermore, the possibility of in-house production of Galium-68 may increase its use and improve nuclear medicine logistics.

Despite all advancements, also FAPI PET has its pitfalls^24^. Like FDG, it shows uptake in benign conditions like liver cirrhosis and bone cysts, which must be considered when diagnosing distant metastases^25,26^. In premenopausal breast cancer patients, physiological FAPI uptake in mammary gland tissue may also be observed; however, this uptake is typically symmetric, which can aid in distinguishing physiological from pathological findings^27^.

In addition to its diagnostic usage, molecular imaging biomarkers may be derived from FAPI PET. Metrics like PET uptake values, metabolic tumor volume (MTV) and total lesion FAP (TLF) may have predictive or prognostic value with regards to therapy response and survival outcomes^28^. Several studies already introduced the prognostic value of [^18^F]FDG uptake in patients with BC, including triple negative subtypes^30,31^ (31,32), and FAPI PET biomarkers are beginning to be evaluated^29–31^. For example, Backhaus et al. demonstrated the suitability for evaluating neoadjuvant chemotherapy response, where [^68^Ga]Ga-FAPI-46-PET/MRI outperformed MRI alone^32^. Moreover, FAPI SUV values, tumor-to-background ratios, MTV, and TLF seem to correlate with tumor aggressiveness (Ki67) and grade^33^ offering prognostic potential at different stages of breast cancer^34^. Interestingly, in our evaluation, potential imaging biomarkers differed between [^68^Ga]Ga-FAPI-46 PET and [^18^F]FDG PET. The MTV was higher for [^68^Ga]Ga-FAPI-46 than for [^18^F]FDG PET (***Fig. ******2***), which may be due to the visualization of a different molecular target, the tumor microenvironment instead of tumor cells. A comparable effect may evoke a larger overlap between individual lesions in [^68^Ga]Ga-FAPI-46 PET, possibly explaining why [^18^F]FDG PET detects more separate lesions in most patients (***Fig. ******2***). Of note, the chosen percentage-based threshold to segment lesions may also contribute to differences in lesion count and volume between tracers. Nevertheless, this suggests that [^68^Ga]Ga-FAPI-46 PET provides complementary information, potentially enhancing prognostic imaging biomarkers, as patients with higher FAP-derived tumor volume may reflect a more stromal/desmoplastic phenotype, whereas FDG-positive/FAP-low lesions could represent biologically more aggressive tumor deposits that may be less suitable targets for FAP-directed therapy. Tumor uptake values, however, were not substantially different between both imaging modalities.

Interestingly, [^68^Ga]Ga-FAPI-46 uptake but not [^18^F]FDG uptake showed a strong negative correlation with the CPS score, which evaluates PD-L1 expression. In unresectable locally advanced or metastatic TNBC the CPS score can be used to predict response to and select patients for additional immunotherapy. Different expression levels may depict distinct tumor immune microenvironments^35^. However, only limited data exist on correlation of FAP and PD-L1 expression. FAP may play a role in promoting immune evasion, varying by tumor type. For example, in NSCLC elevated FAP expression may correlate with decreased CD8 + T-cell density, reduced response to PD-1 blockade, and worse progression-free and overall survival^33^. Moreover, in patients with locally advanced esophageal squamous cell carcinoma a positive correlation between [^18^F]FAPI-04 uptake and PD-L1 expression was described^36^. As the availability of CPS score data in our study was limited, future evaluations in larger cohorts are necessary to evaluate this relationship in patients with TNBC.

In addition to diagnostic purpose, FAPI PET opens the opportunity towards theranostic applications in combination with FAP-targeted radionuclide therapy. We found that 25% of patients at follow-up staging a high average SUV_mean_ ≥ 5 (***Fig. ******4***). Noteworthy no generally accepted threshold to evaluate suitability for FAP-targeted radionuclide therapy has been introduced so far and the clinical impact of FDG-positve/FAPI-negative mismatch has not yet been conclusively determined. In this cohort, two patients at follow-up staging would meet the selection criterion for [^90^Y]Y-FAPI-46 therapy of our institution to show an visual SUV_max_ ≥10 in ≥ 50% of tumor lesions without FDG-positive/FAPI-negative mismatch in relevant lesions^16^. Using this protocol a disease control rate of 38% of patients with mostly sarcoma was demonstrated at a low toxicity profile^16^. Notably, we did not treat patients with TNBC so far. However, in literature few applications of FAP-targeted radionuclide therapy were described^9^. Despite the small number of included patients, these studies highlight the feasibility and safety^9^. In a comprehensive literature review including patients with BC, clinical responses were reported in 11/16 patients^9^.

### Limitations

This study faces several limitations that should be considered when interpreting our findings. First, no histopathological gold standard was available to verify tumor lesions and image interpretation was not blinded. Second, systematic evaluation of brain metastases was not performed, because [^18^F]FDG PET field of view did not include the head. Third, the cohort is small, as inclusion was based on a single-center observational study, resulting in limited statistical power and reflecting the inherent constraints of a post-hoc subgroup analysis, including heterogeneous initial versus follow-up staging populations as well as prior and ongoing systemic therapies in the follow-up cohort. This precludes analysis of possible predictive PET biomarkers, leaving this as a focus for future studies. Moreover, the observed inverse correlation between FAPI uptake and CPS score should be interpreted cautiously and requires validation in larger, independent cohorts. Finally, no standardized eligibility criteria for FAP-targeted radionuclide therapy currently exist and the clinical relevance of discordant FDG-positive/FAPI-negative disease remains uncertain. Accordingly, the proposed SUV_mean_ and SUV_max_ uptake categories should be regarded as institutional and exploratory rather than validated selection criteria for TNBC. In addition, the higher spatial resolution of ^18^F compared with ^68^Ga may have influenced lesion detectability and quantitative assessments. It remains to be determined whether ^18^F-labeled FAPI tracers could provide an advantage in this setting in future studies.

## Conclusion

In patients with TNBC [^68^Ga]Ga-FAPI PET performs equally to [^18^F]FDG PET and offers additional complementary information. At follow-up staging 25% of patients had an average tumor SUV_mean_ ≥ 5 and 17% would fulfill our current institutional inclusion criteria for FAP-directed radionuclide therapy, of homogeneous high uptake in the absence of mismatch in relevant lesions, highlighting its theranostic potential in this still difficult-to-treat tumor entity.

## Supplementary Information

Below is the link to the electronic supplementary material.

Supplementary Material 1

## Data Availability

The datasets generated during and/or analysed during the current study are available from the corresponding author on reasonable request.

## Abbreviations

BC: Breast cancer
CAFs: Cancer associated fibroblasts
CD8: Cluster of differentiation 8
CT: Computer tomography
CTx: Chemotherapy
FAP: Fibroblast activation protein
FAPI: Fibroblast activation protein inhibitor
FDG: Fluorodesoxyglukose
HER2: Human epidermal growth factor receptor 2
LA-ESCC: Locally advanced esophageal squamous cell carcinoma
NSCLC: Non-small-cell lung cancer
MRI: Magnetic resonance imaging
MTV: Metabolic tumor volume
PD-1: Programmed cell death protein 1
PD-L1: Programmed death-ligand 1
PET: Positron-emission-tomography
SUV: Standard Uptake Value
TLF: Total lesion FAP
TLR: Tumor-to-liver ratio
TNBC: Triple-negative breast cancer
TNM: TNM Classification of Malignant Tumors
UICC: Union for International Cancer Control
