# Supplementary material for: Diagnostic value of [68Ga]Ga-FAPI-46 PET in comparison to [18F]FDG PET in triple-negative breast cancer and potential for theranostic application
Source: Sci Rep. 2026 Aug 3;16:26039. doi: 10.1038/s41598-026-61253-8 (PMC13490564; doi:10.1038/s41598-026-61253-8)
Supplement: Supplementary file 1 [file 41598_2026_61253_MOESM1_ESM.docx]

SUPPLEMENTARY INFORMATION TO

Diagnostic value of [^68^Ga]Ga-FAPI-46 PET in Comparison to [^18^F]FDG PET in Triple-negative Breast Cancer and Potential for Theranostic Application

Alina Toni Küper^1^, Ieva Ciuciulkaite^1^, Anja Welt^2,3^, Sofia Carrilho Vaz^4,5^, Ilektra Antonia Mavroeidi^2^, Rainer Hamacher^2^, Tristan Demmert^1^, Kim Magaly Pabst^1^, Alexandros Moraitis^1^, Loic Djaileb^6^, Robert Seifert^1,7^, Lale Umutlu^8^, Martin Schuler^2,3^, Ken Herrmann^1,3^, Wolfgang Peter Fendler^1,3^, Sherko Kümmel^9^, David Kersting^1^

^1^ Department of Nuclear Medicine and German Cancer Consortium (DKTK), University Hospital Essen, University of Duisburg-Essen, Essen, Germany

^2^ Department of Medical Oncology, West German Cancer Center, University Hospital Essen, University of Duisburg-Essen, Essen, Germany

^3^ National Center for Tumor Diseases (NCT), NCT West, Essen, Germany

^4^ Department of Nuclear Medicine and Radiopharmacology, Champalimaud Clinical Center, Champalimaud Foundation, Lisbon, Portugal

^5^ Department of Radiology, Leiden University Medical Center, Leiden, The Netherlands.

^6^ LRB, Nuclear Medicine Department, CHU Grenoble Alpes, INSERM, Université Grenoble Alpes, Grenoble, France.

^7^ Department of Nuclear Medicine, University Hospital Bern, University of Bern, Bern, Switzerland

^8^ Institute of Interventional and Diagnostic Radiology and Neuroradiology, University Hospital Essen, University of Duisburg-Essen, Essen, Germany

^9^Breast Unit Essen, Kliniken Essen-Mitte, Essen, Germany

**Details on imaging protocol**

Before PET acquisition, a contrast-enhanced whole-body CT scan was performed if not clinically available within 4 weeks before the examination date; otherwise, a low-dose CT scan without application of contrast medium was acquired and used for attenuation correction and anatomic localization of PET uptake. For same-day imaging, a minimum interval of 4 hours between tracer administrations was maintained, with the gallium-labeled tracer administered first to avoid potential artifacts affecting the second scan*.* PET data were acquired and PET images were reconstructed according to our standard institutional protocols [24,38].

**Details on detection analysis**

Lesions identified by any detection on [^18^F]FDG- or [^68^Ga]Ga-FAPI-46 PET were used to define regions with viable tumor. Patient-based detection rates were calculated defining every patient with any correctly detected tumor region on [^68^Ga]Ga-FAPI-46 PET and/or [^18^F]FDG as PET-positive detection. Patients were considered negative if no tumor region was detected in any imaging. Region-based detection rates were calculated using the total number of regions that were [^68^Ga]Ga-FAPI-46 PET- and/or [^18^F]FDG PET positive as a reference. Patient- and region-based detection rates were compared between [^68^Ga]Ga-FAPI-46 PET and [^18^F]FDG PET. In all comparisons, brain metastases were not included into analysis due to the high physiologic cerebral uptake in [^18^F]FDG PET which could distort the analysis. Moreover, the standard scan field of view of contrast-enhanced PET protocols for cancer imaging in our institution does not comprise the head.

**Details for lesion analysis and quantification**

Total tumor volume was defined as the sum of the volumes of all [^68^Ga]Ga-FAPI-46 and/or [^18^F]FDG PET-positive lesions. In a PERCIST-like approach, lesions with a peak standard uptake value (SUV_peak_) higher than a liver-specific threshold (1.5 x mean + 2 x SD of SUV) were subjected to segmentation [15]. Lesion boundaries were determined using a 41% isocontour approach[16]. Small lesions with a volume of less than 0.5 ml were excluded. Additional foci with lower SUV values were manually included when required, while physiologic uptake was manually excluded. To calculate the patient-based average tumor-to-liver ratio (TLR_mean_ = tumor SUV_mean_/liver SUV_mean_ or TLR_peak_ = tumor SUV_peak_/liver SUV_peak_) the liver SUV_mean_ or SUV_peak_ was determined in a spheric volume-of-interest of 14 mL volume (3 cm in diameter) in the right liver lobe as suggested in PERCIST 1.0 ([15]. To evaluate the patient-based global tracer uptake (average tumor SUV_mean_), the mean SUV in all [^68^Ga]Ga-FAPI-46 PET- and/or [^18^F]FDG PET-positive segmented tumor lesions per patient was calculated.

ABBREVIATIONS

BC: Breast cancer; CAFs: Cancer associated fibroblasts; CD8: cluster of differentiation 8; CT: Computer tomography; CTx: chemotherapy; FAP: Fibroblast activation protein; FAPI: Fibroblast activation protein inhibitor; FDG: Fluorodesoxyglukose; HER2: Human epidermal growth factor receptor 2; LA-ESCC: locally advanced esophageal squamous cell carcinoma; NSCLC: Non-small-cell lung cancer; MRI: Magnetic resonance imaging; MTV: metabolic tumor volume; PD-1: Programmed cell death protein 1; PD-L1: Programmed death-ligand 1; PET: Positron-emission-tomography; SUV: Standard Uptake Value; TLF: total lesion FAP; TLR: Tumor-to-liver ratio; TNBC: Triple-negative breast cancer; TNM: TNM Classification of Malignant Tumors; UICC: Union for International Cancer Control

| **TABLE S1** Detailed patient characteristics at follow-up staging | | | | | | | |
| --- | --- | --- | --- | --- | --- | --- | --- |
|  | **ID (M/Y)** | **Time to Follow-up Staging** | **Indication** | **Chemotherapy** | | | **Targeted therapy** |
|  |  |  |  | **Neoadjuvant** | **Adjuvant** | **As First line** |  |
| Pat. 11 | Apr/  2021 | 2 Y | New palpable axillary mass | 1 | / | / | / |
| Pat. 12 | Nov/  2021 | 1.5 Y | Suspected pulmonary metastases on conventional imaging | 1 | / | / | / |
| Pat. 13 | Jan/  2019 | 4 Y | Response assessment during targeted therapy | Paclitaxel, Carboplatin | Nab-Paclitaxel^1^ | / | Atezolizumab^1^ |
| Pat. 14 | Jul/  2022 | 7 M | Response assessment during chemotherapy | / | / | Paclitaxel^1^ | Bevacizumab^1^ |
| Pat. 15 | Dec/  2011 | 10 Y | Axillary relapse | TAC | Gemicitabine, Carboplatin; Nab-Paclitaxel, Cisplatin^1^; Cyclophosphamid^2^ | / | Bevacizumab^1;2^ |
| Pat. 16 | Aug/  2013 | 9 Y | Response assessment during chemotherapy | / | FEC; Nab-Paclitaxel^1^; Carboplatin, Gemcitabin^2^; Eribulin; Cyclophosphamid, Vinorebelin, | / | Atezolizumab^1^; Bevacizumab^2^; Sacituzumab -Govitecan |
| Pat. 17 | Jan/  2019 | 3 Y | Response assessment during chemotherapy | EC, Paclitaxel, Carboplatin | Capecitabine, Eribulin | / | / |
| Pat. 18 | 2006 | 16 Y | Suspected local relapse | Paclitaxel, Carboplatin  (for relapse) | / | / | / |
| Pat. 19 | Jun/  2021 | 8 M | Suspected cutaneous metastases | Epirubicin, Cyclophosphamid, Paclitaxel | / | / | Olaparib |
| Pat. 20 | Mar/  2020 | 1.5 Y | Response assessment during chemotherapy | EC, Paclitaxel | Paclitaxel^1^, Eribulin^2^ | / | Atezolizumab^1^; Sacituzumab -Govitecan^2^ |
| Pat. 21 | May/  2021 | 6 M | Multifocal axillary recurrence | Epirubicin, Cyclophosphamid, Paclitaxel, Carboplatin | Capecitabine, Eribulin^1^ | / | Pembrolizumab^1^ |
| Pat. 22 | Jan/  2021 | 1 Y | Restaging after completion of treatment for an early local recurrence | Paclitaxel, Carboplatin; EC | Nab-Paclitaxel^1^ | / | Atezolizumab^1^; Sacituzumab -Govitecan |
| M: month, Y: years, identical superscript numerals within a row indicate therapies initiated simultaneously | | | | | | | |

| **TABLE S2** Detection Rates Stratified by Scan Day | | | | | | |
| --- | --- | --- | --- | --- | --- | --- |
| Initial Staging (10 patients) | | | | | | |
|  | **Different-Day Imaging**  ( 5 patients) | | | **Same-Day Imaging**  (5 patients) | | |
| **Region** | n | | | n | | |
|  | total | FAPI | FDG | total | FAPI | FDG |
| Breast tumor | 5 | 5  (100%) | 4 (80%) | 5 | 5 (100%) | 5 (100%) |
| Regional lymph node metastases | 4 | 2  (50%) | 4 (100%) | 4 | 4  (100%) | 4  (100%) |
| Distant lymph node metastases | 1 | 1  (100%) | 1(100%) | 0 | 0 | 0 |
| Liver metastases | 1 | 1  (100%) | 0 | 0 | 0 | 0 |
| Bone metastases | 2 | 2  (100%) | 2 (100%) | 0 | 0 | 0 |
| Other metastases | 1 | 1  (100%) | 0(100%) | 0 | 0 | 0 |
| Follow-up staging (12 patients, 1 patient without detectable tumor site) | | | | | | |
|  | **Different-Day Imaging**  ( 3 patients) | | | **Same-Day Imaging**  (9 patients) | | |
| Breast tumor | 2 | 2  (100%) | 2  (100%) | 4 | 4  (100%) | 4  (100%) |
| Regional lymph node metastases | 1 | 1  (100%) | 1  (100%) | 7 | 7  (100%) | 7  (100%) |
| Distant lymph node metastases | 1 | 1  (100%) | 1  (100%) | 5 | 5  (100%) | 5  (100%) |
| Liver metastases | 2 | 2  (100%) | 1  (50%) | 2 | 1  (50%) | 2  (100%) |
| Bone metastases | 1 | 0 | 1  (100%) | 2 | 2  (100%) | 2  (100%) |
| Other metastases | 3 | 3  (100%) | 3  (100%) | 7 | 7  (100%) | 7  (100%) |

FIGURE S1

Boxplot representation of mean SUV_max_ values from tumor-affected regions comparing [^68^Ga]Ga-FAPI-46 and [^18^F]FDG PET uptake, separately, in initial (a) and follow-up stagings (b). The boxes represent the interquartile ranges, while whiskers indicate 1.5 times the interquartile range and lines median values. Outliers are indicated as separate data points.
